# Advances in Immunological Methods for the Detection of *Escherichia coli* O157:H7: A Review

**DOI:** 10.3390/s26061894

**Published:** 2026-03-17

**Authors:** Linqing Zou, Chang Xue, Mingyu Tao, Qin Ouyang, Cunzheng Zhang

**Affiliations:** 1 School of Food and Biological Engineering, Jiangsu University, Zhenjian 212013, China; zlq05132025@163.com (L.Z.); 15357630100@163.com (C.X.); oyqyf@163.com (Q.O.); 2 Jiangsu Key Laboratory for Food Quality and Safety-State Key Laboratory Cultivation Base of Ministry of Science and Technology, Institute of Food Safety and Nutrition, Jiangsu Academy of Agricultural Sciences, Nanjing 210014, China; 15862049379@163.com; 3 School of Life and Health Sciences, Environmental Engineering, Hefei University, Hefei 230601, China

**Keywords:** *E. coli* O157:H7, LFIA, ELISA, immunosensors, food safety

## Abstract

**Highlights:**

**What are the main findings?**

- Comprehensive overview of current immunological methods for *E. coli* O157:H7 detection.
- Integration of nanotechnology and bioengineering for enhanced performance.

**What is the implication of the main finding?**

- Future perspectives for food safety monitoring.

**Abstract:**

*Escherichia coli* O157:H7 (*E. coli* O157:H7) is a highly virulent foodborne pathogen with an extremely low infectious dose, making its rapid and accurate detection in food and environmental samples critically important. In recent years, significant progress has been made in immunological techniques for the rapid identification of *E. coli* O157:H7. This review systematically summarizes advances in immunological methods for the detection of *E. coli* O157:H7 over the past decade, focusing on lateral flow immunoassays (LFIA), enzyme-linked immunosorbent assays (ELISA), immunosensors (optical and electrochemical), and nanobody-based technologies. Key aspects such as detection principles, specificity, antibody types (monoclonal, polyclonal, nanobodies), signal readout mechanisms, and applicability to different sample matrices are compared. Performance parameters, including limit of detection (LOD), specificity, detection time, and matrix compatibility, are summarized to evaluate the advantages and limitations of each method. Furthermore, international food safety standards and regulations (ISO 16654, FDA BAM, USDA) are reviewed to highlight the practical and regulatory requirements of these techniques. On this basis, the role of immunological detection technologies in on-site rapid testing is discussed, with a focus on improvements in sensitivity, specificity, and practicality. Finally, future directions are outlined, including multiplexed assays, integration with molecular biology techniques, and engineering applications of nanobody and recombinant technology.

## 1. Introduction

*E. coli* O157:H7 is the prototypical serotype of Shiga toxin-producing *E. coli* (STEC). With an infectious dose as low as 10–100 cells, it frequently causes severe diseases such as hemorrhagic colitis and hemolytic uremic syndrome, posing a major threat to public health [1]. Contamination of food products (e.g., beef, fresh vegetables, dairy) and environmental water by *E. coli* O157:H7 can lead to large-scale foodborne outbreaks, underscoring the urgent need for rapid, sensitive, and specific detection methods [2]. Conventional detection relies primarily on selective enrichment culture combined with biochemical or serological confirmation, which, although accurate, typically requires 2–3 days and is insufficient for timely warning of low-dose contamination. In recent years, nucleic acid amplification techniques (e.g., PCR) have improved sensitivity but still depend on laboratory instrumentation and are unsuitable for on-site use. By contrast, immunological methods based on antigen–antibody specific recognition offer distinct advantages such as rapidity, ease of operation, and field applicability, making them powerful tools for food safety monitoring [3]. Immunoassays target specific antigens of *E. coli* O157:H7 (e.g., O157 somatic polysaccharide antigen, H7 flagellin, or Shiga toxins), enabling direct enrichment and detection of the pathogen either after enrichment or, in some cases, without enrichment.

With advances in biotechnology and materials science, the past decade has witnessed the emergence of various novel immunological detection strategies. For example, the incorporation of nanomaterials has significantly enhanced signal output and improved the sensitivity of lateral flow assays, while the integration of immunomagnetic separation with automation and microfluidics has markedly reduced sample pretreatment time [4]. The development of high-affinity monoclonal antibodies and nanobodies has further improved the specificity and stability of ELISA and immunosensor platforms [5], offering the potential to achieve true enrichment-free rapid detection. Meanwhile, international standards and regulatory agencies have begun evaluating and adopting validated immunoassays as screening tools. For instance, ISO 16654 mandates the use of immunomagnetic beads to enrich *E. coli* O157 from food enrichment cultures prior to isolation [6], while official laboratory methods from the U.S. FDA and USDA include AOAC-approved immunoassays as optional screening steps to expedite the identification of presumptive positives. Collectively, immunological methods serve as rapid “first-line screening” tools in food safety regulation: although their results typically require confirmation by culture or molecular methods, they substantially enhance regulatory efficiency.

**Figure 1:**
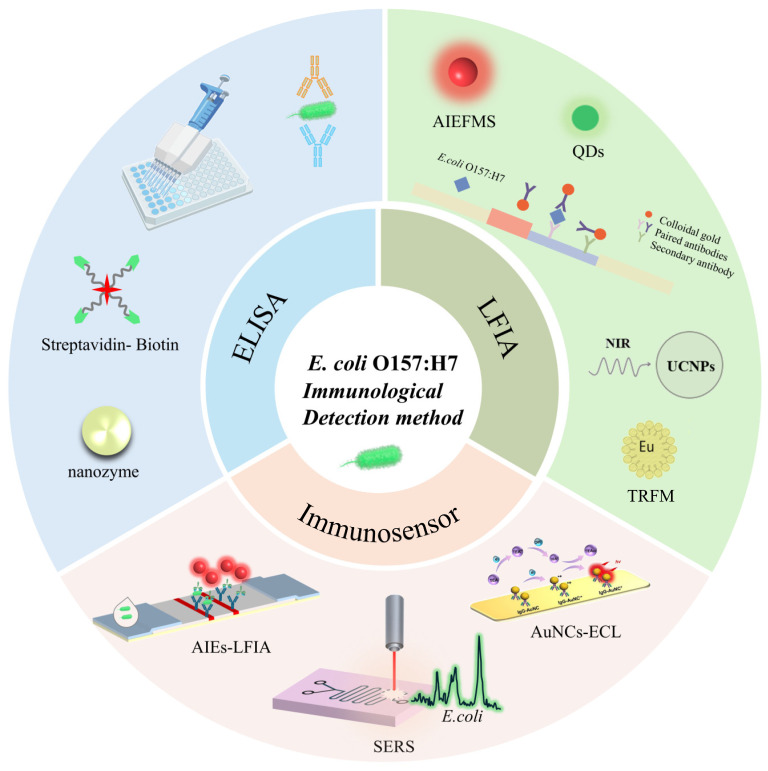
Schematic overview of immunological methods for *E. coli* detection. Abbreviations: *Escherichia coli, E.coli*; ELISA, enzyme-linked immunosorbent assay; LFIA, lateral flow immunoassay; AIEFMS, aggregation-induced emission fluorescent microspheres; QDs, quantum dots; NIR, near-infrared; Eu, europium; UCNPs, upconversion nanoparticles; TRFM, time-resolved fluorescence microscopy; AIE, aggregation-induced emission; SERS, surface-enhanced raman scattering; AuNCs, gold nanoclusters; ECL, electrochemiluminescence; * denotes the excited state, + indicates a positive charge, and • represents a radical species. ECL, electrochemiluminescence.

This review provides a critical overview of recent advances and applications of major immunological detection techniques for *E. coli* O157:H7 in food and environmental samples (see Figure 1). The principles and performance of these methods are compared, and their roles within regulatory testing workflows are discussed in the context of international standards. Table 1 provides a summary of performance parameters across different immunoassay platforms. Finally, the strengths and limitations of current rapid immunological detection technologies are critically evaluated, and future directions are proposed.

## 2. Conventional Immunoassays and Their Improvements

Traditional immunological methods, such as enzyme-linked immunosorbent assay (ELISA) and lateral flow immunoassay (LFIA), remain widely used in the detection of *E. coli* O157:H7 due to their established platforms and broad application basis.

### 2.1. Enzyme-Linked Immunosorbent Assay

ELISA is one of the most extensively applied immunoassays in food safety testing, encompassing four major formats: direct, indirect, sandwich, and competitive assays. The core principle involves immobilizing specific antigens or antibodies on a solid-phase carrier, enabling specific antigen–antibody interactions to capture and detect unknown targets. Detection is subsequently achieved through enzyme-mediated substrate conversion, generating a measurable signal [8]. Given the abundance of antigenic sites on *E. coli* O157:H7, sandwich ELISA is typically employed. ELISA offers advantages including simplicity, low cost, and high throughput screening capability. However, traditional ELISA suffers from lengthy incubation steps (often several hours), labor-intensive operation, and limited sensitivity. Research over the past decade has focused on reducing detection time and improving sensitivity. Three main strategies have been explored:

(1) Increasing the enzyme payload to enhance catalytic efficiency. Conventional antibodies can typically conjugate only 2–3 horseradish peroxidase (HRP) molecules, limiting signal amplification [15]. Biotin-streptavidin systems have been introduced to enhance detection sensitivity. Streptavidin (SA) is a tetrameric protein, with each subunit capable of binding one biotin molecule. This binding is rapid, stable, and unaffected by extreme conditions, providing a reliable foundation for signal amplification. Biotin is covalently linked to the antibody of interest, forming a biotinylated probe. SA can then be conjugated with signal reporter molecules such as enzymes, fluorophores, or colloidal gold to form complexes. Ultimately, each biotinylated probe can bind multiple SA -signal molecule complexes, achieving geometric amplification of the signal [16]. For instance, Guo et al. [17] utilised a biotin–streptavidin amplification system for highly sensitive *E. coli* O157:H7 detection, with the limit of detection being 1.08 × 10^2^ CFU/mL in pure culture. However, as a single SA molecule can bind only four biotin molecules, poly-HRP (PolyHRP) conjugates have been employed to further amplify signals. PolyHRP is a supermolecular polymer of HRP containing up to 400 enzyme molecules at maximum that can be conjugated with various ligands and receptors [18]. Zhang et al. [19] substituted SA-PolyHRP for SA-HRP, significantly improved detection sensitivity, achieving limits of detection (LOD) of 1.4 × 10^4^ CFU/mL for *E. coli* O157:H7, with sensitivity enhancements of 7.86-fold.

(2) Utilizing nanozymes as enzyme substitutes to enhance catalytic efficiency. Compared with natural enzymes, nanozymes exhibit superior environmental tolerance and stability. Wang et al. [20] applied PdRu bimetallic nanozymes as HRP substitutes in ELISA for *E. coli* O157:H7 detection, achieving a 288-fold improvement in sensitivity over conventional HRP-based ELISA. Similarly, Wang et al. [21] used Pd@Pt nanozymes in ELISA, obtaining a 100-fold lower LOD compared with traditional formats.

(3) Incorporating novel signal output elements to enhance catalytic efficiency. Fluorescent nanomaterials and related biosensor platforms have enhanced pathogen detection sensitivity by up to three orders of magnitude, with some systems capable of detecting single bacterial cells. For example, Xue et al. [22] developed a portable biosensor utilising immunomagnetic beads and quantum dots, capable of sensitively detecting *E.coli* O157:H7 at concentrations as low as 14 CFU/mL within two hours.

(4) Enhancing detection sensitivity through the introduction of nanobodies. Nanobodies are variable domains of heavy-chain antibodies (VHH) derived from camelids and represent the smallest known functional antigen-binding fragments (~15 kDa) [23]. They offer several outstanding features, including small molecular size, high stability (resistant to heat and pH extremes), excellent solubility, and low production cost [24,25]. Compared to conventional antibodies, nanobodies can recognize conformation-sensitive or partially hidden epitopes on bacterial surface antigens. Their remarkable refolding capacity after thermal or chemical denaturation makes them particularly suitable for on-site screening applications. For instance, He et al. [5] immunized alpacas to generate nanobodies specifically targeting *E. coli* O157:H7, and subsequently developed a sandwich immunoassay in combination with polyclonal antibodies, achieving a detection limit (LOD) of 8.7 × 10^3^ CFU/mL. Similarly, Xue et al. [26] identified two nanobodies with distinct epitope-binding specificities via phage display screening. By pairing monoclonal antibodies as capture elements with the nanobodies as tracers, they established a phage-mediated triple-antibody sandwich immunoassay that achieved an LOD of 1.89 × 10^3^ CFU/mL, demonstrating no significant cross-reactivity with other bacterial species and achieving a 41.8-fold sensitivity improvement compared with conventional mAb-based sandwich assays.

Despite continuous improvements, ELISA technology remains constrained by its time-consuming and labor-intensive operational procedures (typically requiring 2 to 6 h), and matrix interference from food components necessitates extensive sample pretreatment. Its reliance on specialized personnel and dedicated equipment further limits the applicability of this technology for rapid on-site testing. Moreover, its sensitivity and specificity are lower than those of other methods, with the detection limit of conventional ELISA being approximately 10^4^ CFU/mL—far below the low pathogenic dose of O157:H7 (10–100 cells). This unmet demand has driven the development of LFIA, which provides a simple, rapid, and user-friendly tool for field screening in food safety.

### 2.2. Lateral Flow Immunoassay

**Figure 2:**
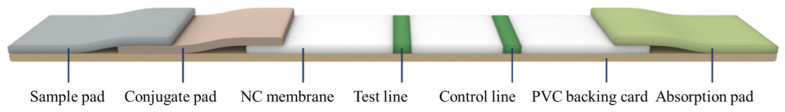
Diagram of the construction of the test strip.

LFIA is a rapid and user-friendly technique that integrates immunological recognition with chromatographic separation [27]. Its core principle relies on immobilizing specific antibodies or antigens onto a nitrocellulose (NC) membrane. When a test sample migrates along the membrane via capillary action, the target analyte interacts with the immobilized antibody or antigen, leading to aggregation of signal labels and generating either colorimetric or fluorescent signals for qualitative detection of the analyte [28]. For the detection of *E. coli* O157:H7, this method typically employs antibodies specific to surface antigens (e.g., lipopolysaccharides or flagellar proteins) immobilized onto an NC membrane. Given the extremely low infectious dose of O157:H7 (as few as 10–100 cells), the sensitivity of conventional LFIA is often insufficient, necessitating the integration of signal amplification strategies—such as the use of nanomaterials or enzymatic reactions—to achieve reliable detection. Additionally, the complexity of food matrices requires that sample pretreatment steps be considered in the assay design to minimize matrix interference and ensure accurate identification of the target pathogen. Figure 2 is an assembly diagram of the test strip. Compared with ELISA, LFIA eliminates time-consuming blocking and washing steps, and the results can be interpreted by the naked eye, making it particularly suitable for on-site rapid testing.

**Figure 3:**
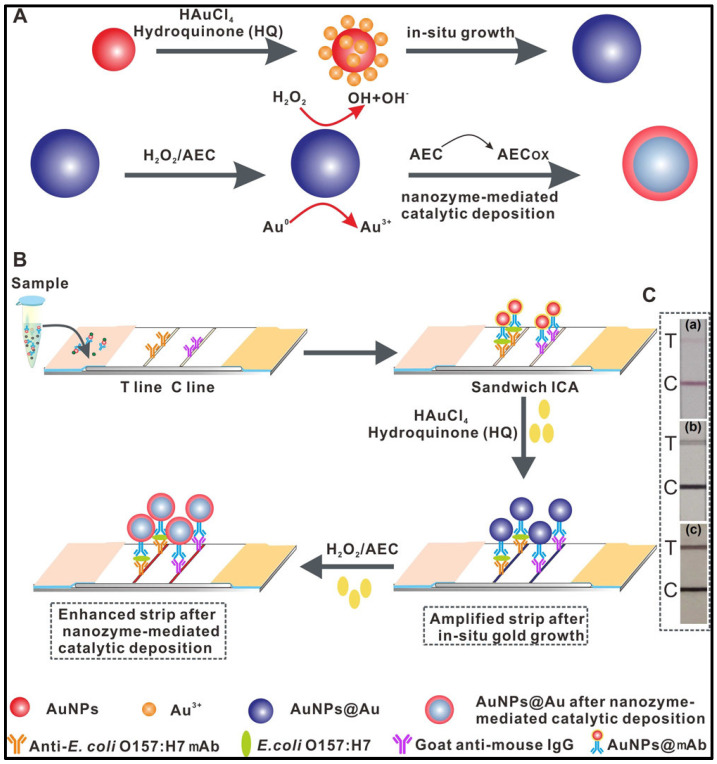
Schematic of the Cascade Signal Amplification Strategy Based on the AuNP-ICA Platform for Ultrasensitive Detection of *E. coli* O157:H7 in Milk. (**A**) Principle and Analysis of the Enhanced AuNP-ICA Method for *E. coli* O157:H7 Detection Using the Proposed Two-Step Cascade Signal Amplification; (**B**) Proposed Two-Step Cascade Signal Amplification Immunoassay; (**C**) 104 CFU/mL of *E. coli* O157:H7 Was Tested Using the Three Strips with or without Signal Amplification; “a” Stands for Conventional AuNP-ICA Strip, “b” Stands for Amplified Strip after in Situ Gold Growth, and “c” Stands for Enhanced Strip after Nanozyme-Mediated Catalytic Deposition Figures are adapted from [30] and reproduced with permission from the American Chemical Society, Copyright © 2020.

Recent advances have significantly improved the sensitivity of LFIA through the incorporation of novel nanomaterials, nanobodies and signal amplification strategies. For instance, Shao et al. [29] constructed a dual-mode colorimetric and fluorescent LFIA strip for the sensitive detection of *E. coli* O157:H7 using dopamine-modified gold nanoparticles (AuNPs), achieving a LOD of 9.06 × 10^1^ CFU/mL, which was 46-fold lower than that obtained using conventional citrate-reduced 40 nm AuNPs. Similarly, Fu et al. [30] proposed an innovative signal amplification strategy by combining in situ growth of colloidal gold with nanozyme-mediated catalytic deposition, yielding a dual-signal enhanced LFIA strip with an LOD of 1.25 × 10^1^ CFU/mL. This approach enabled single-pathogen detection and demonstrated a 400-fold increase in sensitivity compared with traditional AuNP-based LFIA (LOD 5 × 10^3^ CFU/mL). The schematic principle of this strategy is illustrated in Figure 3. In another study, Zhang et al. [10] developed bimetallic Ag–Au sea-urchin-like hollow nanospheres as signal labels for dual-mode LFIA (colorimetric and photothermal), achieving LODs of 2.48 × 10^3^ and 5.5 × 10^2^ CFU/mL, respectively.

These findings demonstrate that LFIA has become a widely applied method for the rapid and sensitive detection of foodborne pathogens. In recent years, the development of advanced nanolabels, the design of multiplex detection systems, and the integration of smartphone-based quantitative analysis have further driven LFIA toward qualitative leaps in performance, significantly enhancing its applicability in food safety monitoring. Despite these advances, traditional colloidal gold immunochromatographic assays (LFIA) for detecting *E*. *coli* O157:H7 still face several inherent limitations that constrain their practicality. The primary challenge lies in insufficient sensitivity and the semi-quantitative detection: conventional colloidal gold-based LFIA typically achieves only a visual detection limit of 10^4^–10^5^ CFU/mL, which is inadequate for detecting low infectious doses of O157:H7. Another critical issue is the non-oriented immobilization of antibodies, which results in inefficient antigen binding and can reduce sensitivity by nearly an order of magnitude compared to optimally oriented forms [31]. Matrix interference in complex food samples often causes non-specific adsorption and background autofluorescence, compromising accuracy. These limitations underscore the need for continued innovation in signal amplification strategies, antibody engineering, and quantitative readout systems.

Compared to these emerging technologies still under development, certain commercial immunoassays have become established industry standards, providing benchmarks for robustness and reliability that next-generation methods must meet. For instance, the [e.g., VIDAS^®^ UP *E. coli* O157 (ECPT)] assay represents a widely adopted automated solution. This method, based on enzyme-linked fluorescent assay (ELFA) technology, has received validation from AOAC International (e.g., Performance Tested Method 060903) and certification by AFNOR according to ISO 16140 standard [32]. In terms of performance metrics, it demonstrates high specificity and sensitivity, with a limit of detection (LOD) capable of identifying 1 CFU in a 25 g food sample following an enrichment period of approximately 8–24 h. The validation studies confirmed a relative sensitivity and specificity of >98% compared to the ISO reference method. Such validated assays serve as a critical benchmark for evaluating the practical utility of emerging immunosensing technologies [33].”

#### 2.2.1. Traditional Labeling Material

Colloidal gold nanoparticles (AuNPs) are the most widely used signal labels in LFIA due to their well-established synthesis protocols and excellent optical properties. Conventional AuNP-based LFIA typically employs citrate-reduced AuNPs (Cit-AuNPs, −35.4 mV), in which the nanoparticle surface is stabilized by strongly ionized citrate ligands, generating a negatively charged surface [34]. The optical properties of AuNPs are largely determined by particle size, with different diameters yielding distinct colors. By adjusting the molar ratio of sodium citrate to chloroauric acid, the nucleation and growth processes of AuNPs can be finely tuned, enabling the preparation of nanoparticles with controlled diameters to meet diverse detection requirements [35]. Compared with other labeling materials, AuNPs exhibit distinct advantages, including biocompatibility, stability, easy visualization, simple preparation, and low cost, making them suitable for large-scale production. Consequently, they have been widely applied in clinical diagnostics, environmental monitoring, and food safety testing [36].

For example, Song et al. [9] developed a sandwich-type multiplex AuNP-LFIA strip for the simultaneous detection of Shigella spp. and *E. coli* O157:H7, with direct detection limits of 10^6^ CFU/mL for both pathogens. After pre-enrichment (10 h for bread and milk, 8 h for jelly), the LOD was significantly improved to 4 CFU/mL. Despite their favorable stability and visual readability, conventional AuNPs have limitations in high-sensitivity assays due to their relatively low molar extinction coefficients and batch-to-batch variability. To overcome these challenges, increasing attention has been directed toward optimizing AuNP size and morphology, as well as coating AuNPs with noble metals or biocompatible shells to enhance light absorption and scattering, thereby improving extinction coefficients. For instance, Zhang et al. [37] demonstrated that surface ligands of AuNPs significantly influence the dynamic adsorption behavior of antibodies. Compared with strongly ionized nanoparticles, weakly ionized AuNPs coated with weak ligands displayed markedly improved antibody adsorption capacity and activity. This strategy enabled AA-AuNPs LFIAs to achieve a 10- to 100-fold increase in sensitivity across multiple assays, highlighting its broad applicability.

#### 2.2.2. Novel Labeling Material

In the past decade, the performance of LFIA has also been significantly enhanced by the development of novel labeling materials. Quantum dots (QDs), owing to their unique optical properties—including broad excitation spectra, narrow emission spectra, high fluorescence quantum yield, strong photostability, resistance to photobleaching, and good biocompatibility when conjugated with biomolecules [38]—have been extensively applied in LFIA. For example, Qiao et al. [39] developed a QD-based paper device for the visual and quantitative detection of *E. coli* O157:H7, integrating immunomagnetic separation with nanoparticle dissolution-triggered signal amplification. This platform achieved a visual detection limit as low as 500 CFU/mL due to its high capture efficiency and effective signal enhancement. Similarly, time-resolved fluorescent nanobeads (TRFNs) have attracted attention because their long fluorescence lifetimes or anti-Stokes shifts effectively minimize autofluorescence from sample matrices, thereby achieving exceptionally high signal-to-noise ratios and sensitivity [40]. However, it should be noted that there are challenges with TRFN, such as: the need for dedicated time-resolved measurement instruments, which increases testing costs and complexity; despite the use of time-resolved technology, interference from background autofluorescence may still occur in complex food matrices; and the stability of fluorescent dyes under long-term storage or repeated testing [41]. Upconversion nanoparticles (UCNPs), which emit visible light under near-infrared (NIR) excitation, exhibit additional advantages such as resistance to photobleaching and minimal background interference. These properties make them highly suitable for bioimaging and analytical detection [42,43]. UCNP-based assays have already been widely applied in food safety and quality monitoring [44]. Despite these advantages, the practical application of UCNPs in food safety monitoring is still hindered by several challenges. For instance, relatively low luminescence efficiency under certain excitation conditions; complexity and batch-to-batch variability in surface functionalization for biomolecule conjugation; and potential concerns regarding long-term toxicity and biocompatibility for in vivo applications, which indirectly affect their regulatory acceptance in food analysis [45].

However, these fluorescent materials are often affected by aggregation-induced quenching (ACQ), where intermolecular π–π stacking or other non-radiative pathways generate radical conjugates, resulting in reduced fluorescence in the aggregated state. In contrast, aggregation-induced emission luminogens (AIEgens) exhibit low fluorescence in the dissolved state but emit strong fluorescence upon aggregation, effectively overcoming the ACQ effect [46]. In the early 21st century, Tang Benzhong’s group discovered aggregation-induced emission (AIE), where molecules fluoresce brighter when aggregated [47]. AIE fluorescent microspheres (AIEFMs), formed from AIE dyes, exhibit high fluorescence by restricting intramolecular motion. Unlike conventional fluorophores with small Stokes shifts, AIEFMs have large Stokes shifts that minimize spectral overlap, reduce autofluorescence, and enhance detection sensitivity and accuracy [48]. Another limitation of conventional dyes is photobleaching. Although QDs are more photostable than organic fluorophores, they may still undergo fluorescence decay due to oxidation in air or aqueous environments. In contrast, AIE molecules maintain structural stability during excitation by restricting intramolecular motions in the aggregated state, allowing them to sustain high fluorescence intensity under prolonged or repeated excitation [49]. This superior photostability makes them particularly suitable for long-term observation or repeated detection scenarios. Currently, AIEFMs-based nanoprobes have been widely applied in immunochromatographic assays (ICA) for the detection of foodborne pathogens, pesticides, and biomarkers. For example, Zhang et al. [11] developed a dual-readout AIEFMs-LFIA for *E. coli* O157:H7 with a LOD of 3.06 × 10^2^ CFU/mL. Similarly, Feng et al. [50] customized an AIEgen-Based Molecular Signaling Tags Combined Microfluidic Chip for POCT Viable *E. coli* O157:H7. Significantly enhanced sensitivity (*E. coli* O157:H7: 45 CFU/mL) and reduced total testing time (45 min). Collectively, these findings highlight the broad application prospects of AIEFMs in ICA. Table 2 provides a summary of performance comparison of newly developed LFIA technologies for the detection of *E. coli* O157:H7 in recent years.

**Figure 4:**
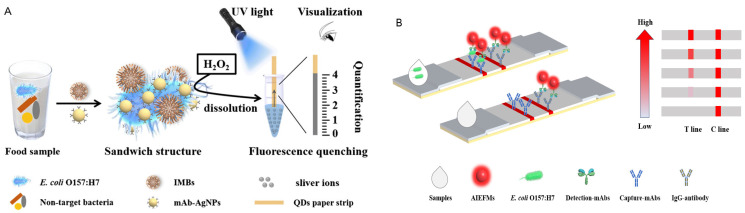
(**A**) Graphical illustration of visual and quantitative detection of *E. coli* O157:H7 by coupling immunomagnetic capture and quantum dot-based paper strip. Figures are adapted from [39] and reproduced with permission from Springer, Copyright © 2021. (**B**) AIEFMS-ICA detection schematic for *E. coli* O157:H7.

Figure 4 shows two representative strategies: one combines immunomagnetic capture with quantum dot-based strips (Figure 4A), and the other uses AIEFMS-ICA for the detection of *E*. *coli* O157:H7 (Figure 4B).

## 3. Immunosensors

Immunosensors are analytical devices that convert antigen–antibody specific recognition events into measurable physical or chemical signals. Typically, they consist of three main components: a recognition element (antibody), a transducer, and a signal processing system [51]. Based on the principle of the transducer, immunosensors can be categorized into electrochemical, optical, piezoelectric, and other types. The integration of nanotechnology has greatly advanced its development, leading to revolutionary improvements in sensitivity, response time, and miniaturization.

### 3.1. Electrochemical Immunosensors

**Figure 5:**
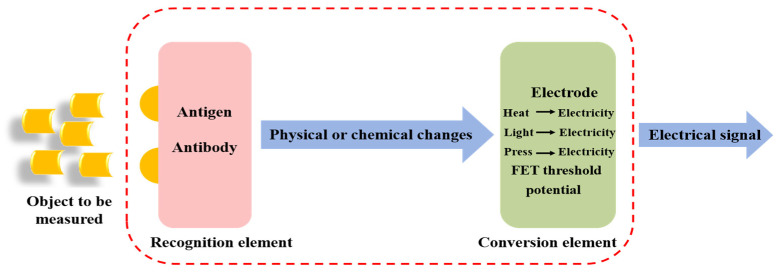
Principle of Operation of Electrochemical Immunosensors.

Electrochemical biosensors operate by immobilizing specific biorecognition elements (e.g., antibodies, enzymes, aptamers) onto the surface of an electrode. During antigen–antibody specific recognition, the target analyte binds to the immobilized recognition element, producing signals that are subsequently converted into electrochemical readouts, enabling qualitative or quantitative detection of the target analyte [52,53]. Based on this, electrochemical immunosensors exhibit higher specificity and selectivity compared with other electrochemical biosensors. However, despite these advantages, electrochemical immunosensors are not without limitations. Their single-use nature means they cannot be recycled, leading to increased costs for frequent testing. Furthermore, they often suffer from a short shelf life and are restricted to a limited temperature range, which poses challenges for storage and transportation. Additionally, these sensors are prone to unstable voltage and unstable current during operation, which can compromise the reliability and reproducibility of the electrochemical readouts [54]. The basic principle of operation of electrochemical immunosensors is shown in Figure 5. Depending on the type of electrochemical change detected through the biorecognition event, electrochemical biosensors can be divided into four categories: impedance, amperometric, conductometric, and potentiometric [55]. Among them, amperometric immunosensors are currently the most extensively studied and widely applied.

(1) Impedimetric Immunosensors.

Impedimetric immunosensors employ electrochemical impedance spectroscopy (EIS) to detect changes in electrode impedance values that arise from the specific binding of immune complexes or enzyme–substrate reactions on the electrode surface [55]. To amplify impedance signals, researchers have modified electrodes with poorly conductive polymers or biomacromolecules, or by capturing non-conductive nanoparticles. For example, Malvano et al. [56] developed a label-free impedimetric immunosensor for the detection of *E. coli* O157:H7, applying different immobilization strategies for monoclonal anti-*E. coli* antibodies. They emphasized the advantages of oriented immobilization and the use of dendritic polymers, which increased the density of immobilized antibody units and thereby enhanced sensitivity. Furthermore, activated ferrocene was employed as an electron transfer mediator to improve the electrochemical performance of the system, achieving a detection limit as low as 3 CFU/mL. When applied to milk and meat samples, the immunosensor yielded results consistent with those obtained by ELISA, demonstrating excellent applicability for food analysis.

(2) Amperometric Immunosensors.

Amperometric immunosensors combine immunological techniques with electrochemical detection and typically employ enzymatic or electroactive labels. Commonly used enzymes include alkaline phosphatase, horseradish peroxidase, lactate dehydrogenase, glucose oxidase, penicillin oxidase, and urease [49]. Label-free amperometric immunosensors are widely used for real-time monitoring of antigen–antibody interactions, as binding events induce changes in electrode current density. Potential pulse techniques are the most commonly applied detection approach [57]. To enhance sensitivity, efforts have focused on electrode modification and signal amplification. Nanomaterials such as gold nanoparticles (AuNPs) [55], carbon nanotubes (CNTs) [58], and graphene [59] have been employed to increase electrode surface area, improve antibody immobilization, and accelerate electron transfer. Nidhi et al. [60] further demonstrated that nickel oxide (NiO), acting as a specialized antibody carrier, facilitated accelerated charge transfer and enhanced target capture efficiency, thereby enabling direct detection of *E. coli* O157:H7. Their electrochemical immunosensor exhibited a wide linear detection range (10^1^–10^7^ cells/mL) and an impressively low detection limit of 1 cell/mL, with high selectivity and specificity compared with other bacterial species. Compared with other electrochemical immunosensors, amperometric immunosensors are characterized by simplicity of operation, low cost, high sensitivity, and suitability for miniaturization and array formats. As a result, they have received growing attention and application in medical diagnostics, food safety monitoring, environmental analysis, and industrial production in recent years.

### 3.2. Optical Immunosensors

Optical immunosensors detect changes in optical properties—such as absorption, reflection, fluorescence, or Raman scattering—triggered by immune recognition events. They are characterized by high sensitivity, high throughput capability, and diverse detection modalities [61].

(1) Electrochemiluminescent Immunosensors.

Electrochemiluminescence (ECL) refers to light emission generated through electron transfer in an electrochemical process, followed by radiative relaxation of excited states to ground states. Due to its excellent controllability, low background signal, and high sensitivity, ECL has been widely applied in the detection of pathogens, disease biomarkers, environmental contaminants, and foodborne pollutants [62,63]. Nevertheless, the preparation of antibody-labeled ECL probes and ECL immunoassay (ECLIA) platforms is often complex, hindering further development. Because directly labeled antibodies generally exhibit low sensitivity, numerous elaborate signal amplification strategies have been proposed. For instance, Hong et al. [63] reported a simplified ECL immunosensor using “two-in-one” IgG–Au nanoclusters (IgG-AuNCs) as multifunctional probes. The synthesized IgG-AuNCs not only exhibited excellent ECL properties but also retained the biological activity of IgG, demonstrating their dual functionality as superior ECL emitters and effective biorecognition elements. In addition to ECL-based methods, other electrochemical approaches have also been developed for pathogen detection. For instance, Yin et al. [64] have successfully developed a paper-based near-infrared responsive PEC sensing platform utilising UCNPs@SiO_2_@Ag and carbon-graphene-carbon-nitride (C-g-C_3_N_4_) for the rapid detection of *E. coli* O157:H7. The detection limit is 2 CFU/mL, with a total testing time of just 50 min.

Metal–organic frameworks (MOFs), which are porous crystalline materials self-assembled from inorganic nodes (metal ions or clusters) and organic linkers, have recently been employed in ECL sensor construction. Their large surface area, porous structure, and good biocompatibility allow efficient loading of ECL luminophores or electroactive species [65,66]. For example, Cao et al. [67] utilized a PbZr-MOF-based signal nanotag (sDNA2-AuNPs@PbZr-MOF) in a sandwich hybridization assay on a modified glassy carbon electrode to achieve sensitive electrochemical detection of *E. coli* O157:H7, obtaining a wide linear range from 2.2 to 2.2 × 10^5^ CFU/mL and a low detection limit of 0.80 CFU/mL, along with excellent reproducibility, selectivity, and stability.

(2) Surface Plasmon Resonance (SPR) Immunosensors.

SPR immunosensors are optical biosensing platforms that detect biomolecular interactions by measuring changes in the refractive index near a metal surface [68]. To improve the sensitivity of SPR detection, researchers have developed various signal amplification strategies. One notable approach is the use of localized surface plasmon resonance (LSPR) properties of metallic nanostructures, which can significantly enhance SPR signals. By tuning the shape and size of these nanostructures, the signal amplification effect can be optimized. For instance, Mishra et al. [69] discussed an early-stage detection of detrimental *E. coli* bacteria using an SPR sensor in the NIR region (at a wavelength of 1000 nm). In their work, the sensor structure consists of CaF_2_/Ag/TiO_2_/poly-l-lysine/sensing element. The proposed structure consists of TiO_2_ as a dielectric layer, which improves plasmon excitation and enhances the performance of the SPR sensor. In another study, Shi et al. [70] employed a nanozyme-triggered polymerization amplification strategy to construct a highly sensitive SPR immunosensor. The resulting platform demonstrated a broader linear range, lower detection limit, and excellent stability, underscoring the potential of nanomaterial-assisted amplification in advancing SPR-based immunoassays.

(3) Fluorescence Immunosensors.

Fluorescence immunosensors combine the optical properties of fluorophores with immunorecognition to detect biomolecules. Fluorescence arises when electrons in a fluorophore absorb light of a specific excitation wavelength, transition from the ground state to an excited state, and subsequently return to the ground state while releasing excess energy as light. Different fluorophores have distinct excitation and emission wavelengths [71]. Common strategies involve the use of fluorescent nanomaterials (e.g., quantum dots, carbon dots, aggregation-induced emission nanoparticles), Förster resonance energy transfer (FRET) probes, or fluorescence quenching mechanisms as labeling or signal systems.

For example, Wei et al. [72] developed a novel luminescent immunoassay termed d-AIENPs-LFIA for the detection of *E. coli* O157:H7. By employing ultra-bright red-emissive aggregation-induced emission nanoparticles (AIENPs) as signal amplification probes in LFIA, they achieved a fluorescence quantum yield of 38.7% and superior signal stability. The assay demonstrated excellent sensitivity, enabling detection of *E. coli* O157:H7 within 15 min, with LODs of 396 CFU/mL. FRET-based immunosensors have also been extensively explored. When a donor fluorophore (e.g., QDs) and an acceptor (e.g., AuNPs or quenchers) come into proximity or separate due to immune binding events, energy transfer occurs, resulting in fluorescence quenching or recovery [73]. Wang et al. [74] designed a novel FRET immunosensor for *E. coli* O157:H7 detection in food samples by integrating carbon dots (CDs) as fluorescence donors and covalent organic frameworks (COFs) as acceptors. Specific antibodies against *E. coli* O157:H7 (Ab) were used to link CDs and COFs. Binding of the antibody to *E. coli* O157:H7 disrupted the CD–COF connection, restoring CD fluorescence. This immunosensor exhibited a linear range of 0–10^6^ CFU/mL with a remarkably low LOD of 7 CFU/mL. Similarly, Fang et al. [75] synthesized a porous coordination network (PCN-224) with strong fluorescence enhancement under alkaline conditions, where the fluorescence intensity increased 20.4-fold due to partial restoration of electron cloud density caused by decreased Zr^4+^ content. Considering the strong overlap between the excitation spectrum of PCN-224 and the absorption band of Ag nanoparticles (AgNPs), coating PCN-224 with a Ag layer triggered fluorescence quenching, which was applied in a “turn-off” immunoassay for the sensitive detection of *E. coli* O157:H7. This assay achieved a low LOD of 3.3 × 10^2^ CFU/mL, which was 29.7 times more sensitive than conventional ELISA.

Compared with other bioanalytical methods, fluorescence immunosensors offer several unique advantages: (1) they allow real-time monitoring and point-of-care testing (POCT) with ultrahigh sensitivity and high throughput; This exactly meets the detection requirements for O157:H7, which has an extremely low infectious dose (only 10–100 cells). (2) They can achieve selective recognition and precise quantification of diverse targets through chemical modification or biosynthetic strategies [76]. Nevertheless, fluorescence immunosensors generally require expensive instrumentation, signal conversion components, controlled environments, and skilled operation. Moreover, they often rely on ultraviolet or laser excitation, both of which suffer from limited penetration depth, restricting their applicability in complex sample matrices (e.g., ground beef, milk). These matrix effects necessitate extensive sample pretreatment, which undermines the speed and simplicity that fluorescence immunosensors ideally promise.

(4) Surface-Enhanced Raman Scattering (SERS) Immunosensors.

Surface-enhanced Raman scattering (SERS)-based immunoassays exploit the surface enhancement effects of metallic nanoparticles, combining the ultrahigh sensitivity and spectral selectivity of SERS with the specific recognition ability of antibody–antigen interactions [77]. Over the past decade, SERS has developed rapidly and has been widely applied in analytical science, surface science, and biosciences. When target molecules are adsorbed onto roughened noble metal nanoparticle surfaces or are positioned in close proximity, their Raman signals are dramatically enhanced, with enhancement factors reaching 10^6^–10^14^ [78,79].

In SERS immunoassays, Raman reporter molecules (e.g., rhodamine 6G) are typically adsorbed onto noble metal nanoparticles to generate SERS tags, which are then conjugated to antibodies. Upon binding of the SERS tag to the target pathogen, the characteristic Raman peaks of the reporter molecule can be measured to achieve quantitative detection. With its unique molecular fingerprinting capability, SERS is particularly well suited for multiplex analysis [80]. For example, Zhu et al. [81] developed a novel SERS nanoprobe by integrating silica-encapsulated gold nanoparticles (SEGN) with functionalized magnetic nanoparticles (MNPs) for highly sensitive and specific detection of *E. coli* O157:H7, achieving a detection limit as low as 10 CFU/mL.

Compared with fluorescence immunosensors, SERS offers several advantages [79]: (1) resistance to photobleaching and strong tolerance against autofluorescence interference; (2) significantly narrower Raman spectral peaks compared with fluorescence emission bands, with linewidths as narrow as 4 cm^−1^ (0.3 nm), making SERS ideal for multicomponent analysis; (3) ultrahigh sensitivity, with large SERS enhancement factors enabling even single-molecule detection; and (4) rapid and simple operation, requiring only small sample volumes (typically 1–2 μL), with detection completed within seconds. These features highlight the promising potential of SERS in immunodetection. Nevertheless, two major challenges remain [82]: (a) the development of efficient and reproducible SERS substrates, and (b) the establishment of accurate and reliable data analysis methods for SERS signals.

(5) Colorimetric Immunosensors.

Colorimetric immunosensors are analytical devices that detect and quantify target analytes by translating antigen–antibody specific interactions into visible color changes. Their fundamental principle involves immobilizing antigens or antibodies onto a designated sensing area. When the target antigen or antibody in the sample specifically binds to the immobilized counterpart, the resulting physicochemical changes at the sensor surface trigger a colorimetric response. The degree of color change can then be visually observed or quantitatively measured using spectrophotometry. Owing to their simplicity, low equipment requirements, and suitability for on-site applications, colorimetric immunosensors are particularly advantageous in resource-limited environments.

Among various labeling materials, gold nanomaterials have been most widely used in colorimetric immunosensing because of their ease of preparation, low cost, high sensitivity, and excellent applicability for point-of-care detection [83]. Leveraging the unique optical properties of AuNPs—particularly their localized surface plasmon resonance (LSPR)—antibody-conjugated nanoparticles undergo aggregation or dispersion upon binding to *E. coli* O157:H7, leading to changes in light absorption and scattering and producing visible color variations. For example, Wang et al. [84] synthesized capture antibody-modified magnetic nanoparticles (cMNPs) and detection antibody/HRP co-functionalized AuNPs (dHAuNPs) for targeted enrichment and colorimetric detection of *E. coli* O157:H7. By loading large amounts of HRP on AuNPs for signal amplification, their system achieved high sensitivity (LOD: 1.63 CFU/mL), a short assay time (3 h), and strong anti-interference performance even in real sample analyses.

Nanozymes have also been extensively applied in colorimetric sensors due to their advantages of high stability, low cost, tunable catalytic activity, and facile surface modification [85]. Nanozymes are nanomaterials with intrinsic enzyme-like catalytic activity, combined with unique optical properties, making them promising signal-generating labels in analytical chemistry. Based on their signal transduction mechanisms, colorimetric immunoassays using nanozymes can be broadly divided into three categories: (1) those utilizing the intrinsic optical properties of nanoparticles (e.g., as optical tags in LFIA for naked-eye detection); (2) those relying on nanozyme-catalyzed substrate conversion to generate colorimetric products, analogous to ELISA but with nanozymes replacing natural enzymes; and (3) those in which catalytic products induce optical changes in nanoparticles, altering absorbance or absorption wavelength to produce a colorimetric response [86].

The peroxidase-like activity of nanozymes is the most widely exploited property. By catalyzing the oxidation of chromogenic substrates such as TMB into detectable colored products, nanozymes such as Fe_3_O_4_, CeO_2_, and AuNPs have been shown to significantly enhance detection sensitivity, with greater stability than natural enzymes [87,88]. For instance, Jiang et al. [89] prepared FeCoMOF/Co_3_O_4_@PDA nanozymes that could be directly conjugated with antibodies as probes for *E. coli* O157:H7 detection. They developed an improved ELISA-based dual-mode platform integrating colorimetric sensing with intelligent detection, achieving a wide dynamic range (10^1^–10^8^ CFU/mL) and an ultralow detection limit of 2 CFU/mL.

Despite their significant progress, each single-mode biosensing technology has inherent limitations. Electrochemical biosensors, although highly sensitive, often suffer from poor stability and reproducibility and are susceptible to environmental fluctuations (e.g., temperature and pH). Fluorescence biosensors typically require UV or laser excitation, but UV light has limited penetration depth, which restricts their application in complex sample matrices. To address these challenges and meet the growing demand for early disease diagnosis, dual-mode biosensors have emerged as a promising next-generation solution. Table 3 shows a comparison of the performance of various immunosensor technologies for detecting *E. coli* O157:H7 in recent years.

## 4. Key Technologies and Strategies

The performance of immunological detection methods depends not only on the sensor itself but also on efficient sample pretreatment techniques and high-quality biorecognition elements.

### 4.1. Sample Pretreatment Strategy

(1) Immunomagnetic Separation (IMS)

Food samples are characterized by complex matrices and low concentrations of target pathogens, making direct detection challenging to achieve the required sensitivity. Magnetic separation is a technique that employs magnetic materials to isolate and extract target analytes [102]. Functionalized magnetic beads can form complexes with recognition molecules and target pathogens, enabling rapid solid–liquid separation under an external magnetic field and facilitating subsequent signal detection [103]. Almost all types of immunoassays (ELISA, LFIA, PCR, and various biosensors) can be combined with IMS to form integrated “IMS–detection” platforms, which have become a standard strategy for the detection of trace pathogens. Magnetic nanomaterials, particularly magnetic nanoparticles, have shown remarkable progress in the enrichment of *E. coli* and in nucleic acid purification, establishing themselves as one of the key technologies for rapid and efficient detection [104]. Their surfaces are often modified with polymers such as polyethyleneimine (PEI) [105] to enhance binding affinity with *E. coli*. In addition, magnetic nanoparticles can be conjugated with other biomolecules—including antibodies, antimicrobial peptides [106], bacteriophages, or aptamers [107]—to improve capture specificity for particular bacteria. For instance, Chen et al. [108] developed a dipstick-type signal-amplified immunoassay (DSIA) for rapid, simple, and sensitive monitoring of Salmonella typhimurium and *E. coli* O157:H7. Leveraging CFO-based magnetic enrichment, the DSIA achieved matrix-independent detection with a dynamic range of 10^2^–10^8^ CFU/mL and a detection limit of 10^2^ CFU/mL. Wang et al. [84] developed a highly integrated magnetic separation enzyme-linked immunosorbent assay system for the highly sensitive and rapid detection of *E. coli* O157:H7. This sensing platform exhibits excellent specificity and resistance to interference, with a detection time of 3 h and a detection limit as low as 1.63 CFU/mL.

(2) Filtration-assisted sample preparation (FASP)

FASP has emerged as a promising approach for field-deployable detection. Han et al. developed an integrated platform combining multi-filter preprocessing with a bifunctional linker-based biosensor, capable of isolating *E. coli* O157:H7 from food samples without complex centrifugation or enrichment steps. This system achieved a detection limit of 10^2^ CFU per 25 g of tomato within 2.5 h from sampling to result, while handling sample volumes up to 250 mL—a critical feature for maximizing bacterial capture from heterogeneous food matrices [109]. The FASP approach effectively addresses the dual challenges of removing PCR inhibitors and concentrating target cells, making it particularly suitable for integration with downstream immunological detection systems.

(3) Flow cytometry

Flow cytometry-based approaches with simplified sample preparation have demonstrated remarkable sensitivity for low-level pathogen detection. Williams et al. [110] developed a method incorporating brief non-selective enrichment (6.5 h at 42 °C), centrifugation for cell concentration, and fluorescent antibody labeling, achieving detection of a single viable *E. coli* O157:H7 cell in 25 g of raw spinach within 9 h total time-to-results. This approach proved more sensitive than reference regulatory methods and eliminated the need for plate-based strain isolation, significantly reducing complexity.

### 4.2. Strategies to Improve Antibody and Enzyme Stability

The functional stability and activity of biorecognition elements—both antibodies and enzymes—are critically dependent on their immobilization onto solid supports (e.g., nitrocellulose membranes, nanoparticles, or frameworks) [111]. For antibodies, oriented immobilization strategies that maximize antigen-binding site exposure have proven highly effective. Techniques such as Protein A/G-mediated capture, site-specific conjugation via carbohydrate moieties, or the use of Fc-binding proteins ensure that the antigen-binding fragments (Fab) remain accessible, thereby enhancing both assay sensitivity and long-term storage stability [112]. Similarly, enzyme immobilization has evolved beyond simple physical adsorption; advanced approaches, such as encapsulating enzymes within hydrogen-bonded organic frameworks (HOFs) via biomimetic mineralization [113], provide a protective microenvironment that shields enzymes from environmental stressors (e.g., temperature, pH, proteases) while preserving catalytic efficiency.

Using recombinant technology, engineered antibody fragments (such as single-chain variable fragments, scFvs) or enzymes with enhanced intrinsic stability can be designed. Compared to full-length antibodies, these fragments typically exhibit greater resistance to thermal denaturation and aggregation [114].

Finally, lyophilization with appropriate excipients remains a gold-standard method for long-term preservation without refrigeration. Collectively, these strategies are essential for translating laboratory-based assays into reliable, field-deployable tools for *E. coli* O157:H7 monitoring.

### 4.3. Development of Novel Recognition Elements

The molecular basis of immunoassays lies in the high specificity and affinity of antibody–antigen interactions; thus, antibody performance is critical to assay sensitivity and accuracy. Conventional antibodies, including monoclonal and polyclonal antibodies, have inherent limitations such as long production cycles, high costs, and batch-to-batch variability [115]. Over the past decade, advances in antibody engineering technologies and the use of alternative recognition molecules—such as aptamers and molecularly imprinted polymers—have provided promising solutions to these challenges.

(1) Molecularly Imprinted Polymers (MIPs).

MIPs are a class of polymers with specific recognition capabilities. The core principle of MIPs is the formation of complementary imprinted cavities in the polymer network using template molecules (target molecules), enabling highly specific recognition of the target molecules during detection [116,117]. Specifically, they can form imprinted cavities complementary to the size, shape, and surface characteristics of O157:H7 or its specific biomolecular markers (such as lipopolysaccharides). This allows MIPs to selectively bind to the target pathogen during the detection process. MIPs possess high specificity, excellent chemical and thermal stability, low production cost, and reusability, effectively overcoming the limitations associated with enzymes, antibodies, and aptamers in terms of stability, production costs, and storage conditions [118,119]. These attributes position MIPs as particularly promising candidates for developing robust, field-deployable immunosensors for O157:H7 monitoring in food safety applications. Zhou et al. [120] developed an MIPs-based photoelectrochemical (PEC) sensor with modified single-atom-homojunctions for *E. coli* O157:H7 detection, achieving a linear range of 10–10^8^ CFU/mL and a limit of detection of 3 CFU/mL.

(2) Recombinant Antibodies (rAbs).

Recombinant antibodies are produced by cloning antibody gene sequences into plasmids via recombinant DNA techniques and expressing them in suitable host cells. Compared with polyclonal and monoclonal antibodies, rAbs provide several advantages: high batch-to-batch consistency and reproducibility, scalable and sustainable production without animal use, and easy genetic modification [121]. Technologies such as phage display allow the screening of antibody fragments—such as single-chain variable fragments (scFv) and antigen-binding fragments (Fab)—from gene libraries. These recombinant fragments can be expressed in *E. coli* or yeast systems, ensuring production consistency and facilitating genetic engineering (e.g., tag incorporation) [122].

Phage display antibodies in particular offer unique advantages: they do not require immunization, are faster and simpler to develop, provide direct access to antibody gene sequences, allow greater control over the selection process, and can generate antibodies against antigens with or without natural immunogenicity. Furthermore, the method is accessible to basic molecular biology laboratories, as it does not require high-end instrumentation [123]. For example, Li et al. [124] screened a specific peptide (E2) against *E. coli* O157:H7 using phage display, synthesized a biotinylated version, and conjugated it with streptavidin-coated magnetic beads (MBs) to construct peptide–MBs probes. These probes were then employed for sample enrichment, and a QD-based multicolor fluorescent assay was developed, achieving a detection limit of 10^3^ CFU/mL. To date, however, reports on recombinant antibody-based detection of *E. coli* O157:H7 remain limited.

## 5. Conclusions and Future Perspectives

In summary, immunological detection technologies are playing an increasingly important role in food and environmental monitoring of *E. coli* O157:H7. Lateral flow assays provide convenient and rapid on-site screening; immunomagnetic separation (IMS) significantly enhances sensitivity; ELISA offers reliable quantitative confirmation in laboratory settings; while immunosensors and nanobody-based assays represent the forefront of future technological development. Their common advantage lies in the exploitation of antigen–antibody specific recognition to achieve rapid identification of target pathogens, markedly accelerating detection compared with conventional culture methods. Particularly in large-scale sample screening, immunological assays can substantially reduce the number of samples requiring confirmatory testing, thereby saving valuable time for regulatory agencies to implement timely interventions. Despite current limitations in sensitivity and quantification, these challenges are being progressively addressed through ongoing technological advancements.

Looking forward, the integration of biosensing, advanced materials, and protein engineering is expected to drive immunoassays toward becoming more sensitive, accurate, and multifunctional. Once breakthroughs in critical technologies—such as enrichment-free ultrasensitive detection, multiplex capability, and standardized nanobody applications—are achieved, these methods may expand their scope in food safety monitoring and potentially become mainstream tools for routine detection. The continued innovation and development of immunological detection technologies will be pivotal for safeguarding public health and ensuring food supply chain security.

Future Directions: To overcome existing limitations, future research and development efforts will focus on the following aspects:

1. Component Level: Advancing Recognition Elements

At the core of immunosensor development, continuous innovation in recognition elements is essential for enhancing stability, affinity, and reproducibility.

Nanobody and recombinant antibody applications: Advances in antibody engineering are expected to generate a wider range of nanobodies targeting O157 antigens and virulence factors. These will serve as highly stable, high-affinity probes for ELISA, LFIA, and biosensor platforms, with potential breakthroughs such as room-temperature storage and probe reusability.

2. Performance Level: Enhancing Analytical Capabilities

Building upon improved components, the next level focuses on pushing the boundaries of sensor performance to meet the stringent demands of food safety monitoring.

Mitigate matrix interference issues: IMS is the gold standard for isolating *E. coli* O157:H7 from complex matrices while removing inhibitory compounds. Additionally, simple physical interventions like filtration and appropriate sample dilution, and coating sensor surfaces with blocking agents such as Bovine Serum Albumin (BSA), casein, or polyethylene glycol (PEG), prevent non-specific adsorption of food residues and can also mitigate matrix interference issues in complex samples.

Improving sensitivity: Signal amplification strategies (e.g., catalytic cycles, nanoprobe aggregation) will be explored to further reduce detection limits and shorten, or even eliminate, enrichment steps. For instance, combining CRISPR/Cas with immuno-enrichment to amplify nucleic acid signals holds promise for ultrasensitive detection.

Multiplex detection: Expanding the ability to detect multiple targets simultaneously will greatly enhance practicality. Future platforms may allow a single strip or sensor to identify *E. coli* O157:H7 along with other major STEC serotypes, or even their toxins, thereby providing comprehensive diagnostic results.

3. Platform Level: Toward Integrated and Intelligent Systems

Smart and portable platforms: The integration of simple and efficient sample preparation with immunosensor platforms is a critical prerequisite for true field deployability. Future devices should incorporate multifunctional components—such as immunomagnetic beads for simultaneous target capture and signal generation—directly into portable formats. When coupled with the Internet of Things (IoT) and smartphone-based readout systems, these integrated platforms will enable real-time, on-site detection, immediate data uploading, and digitalized food safety surveillance, empowering non-specialists to perform robust analysis in resource-limited environments.

Hybrid detection strategies: The integration of immunological methods with other advanced technologies will create new modes of detection. Examples include microfluidic chips integrating IMS enrichment, immunoassay, and electronic readout in a single automated process, or workflows combining immuno-capture with mass spectrometry or sequencing for confirmatory identification, thereby balancing speed with accuracy.

Collectively, these directions highlight the significant promise of immunological detection technologies to evolve into faster, smarter, and more reliable platforms, thereby making profound contributions to the future of food safety monitoring and public health protection.

## Figures and Tables

**Table 1:** Advantages and limitations of existing methods for *E. coli* O157:H7 detection.

Methods	Limit of Detection (LOD)	Specificity	Detection Time	Matrix Applicability & Notes	Ref.
Enzyme-Linked Immunosorbent Assay (ELISA)	Typical sandwich ELISA: −10^5^ CFU/mL; −10^4^ CFU/mL with signal enhancement; Paper-based ELISA can reach 10^4^ CFU/mL	Highly specific antibodies, no significant cross-reactivity; detects dead bacteria (detects total antigen)	2–4 h (excluding enrichment culture)	Requires laboratory operation and microplate reader. Suitable for confirmation or quantitative analysis; limited field application.	[7,8]
Lateral Flow Immunoassay (LFIA)	Traditional colloidal gold: 10^4^–10^5^ CFU/mL; Improved fluorescent/nanomaterial labels: 10^2^–10^3^ CFU/mL	High specificity for O157 serogroup; Does not detect non-O157 STEC (requires other methods to distinguish H7 subtype)	10–20 min (without enrichment); Enrichment typically 8–18 h	Enrichment broth, clean water samples can be tested directly; food requires enrichment or pretreatment to reduce matrix interference. Portable, suitable for high-volume initial screening.	[9,10,11]
Optical Immunosensor	Label-free sensing (e.g., SPR): 10^2^–10^3^ CFU/mL level; SERS enhanced: 10^1^–10^2^ CFU/mL	Specificity determined by antibody; complex matrices may cause non-specific signals, requiring appropriate blocking/calibration	Several minutes to tens of minutes	Real-time detection, enrichment-free with expensive instruments. Suitable for clean or simply pre-treated samples; mostly used in research validation for actual food testing.	[12]
Electrochemical Immunosensor	Labeled immune amperometry: −10^2^ CFU/mL; Label-free impedance type: best case 1–10 CFU/mL	High specificity; electrode surface prone to non-specific adsorption from matrix, requires surface blocking and optimized antibody conjugation	5–30 min (excluding possible pre-enrichment)	Devices can be miniaturized for field use. Often combined with immunomagnetic enrichment or microfluidics to improve reliability with complex samples.	[12,13]
Nanobody-based Immunoassay	Nanobody-ELISA: −8.7 × 10^3^ CFU/mL; Nanobody-LFIA: can outperform corresponding IgG LFIA (several-fold sensitivity increase)	Nanobodies have high affinity and strong specificity; stable to heat/pH, reducing risk of false negatives in the field	Same as corresponding format (e.g., ELISA: 2–3 h, LFIA: 10–20 min)	Higher tolerance, suitable for various food matrices. Easily fused with enzymes or labels for novel sensing amplification. Currently in R&D phase, promising prospects.	[14]

**Table 2:** Performance comparison of newly developed LFIA technologies for the detection of *E. coli* O157:H7 in recent years.

Detection Technologies	Labeling Material	Limit Detection LOD (CFU/mL)	Detection Time	Sample Type	Ref.
Multiplex AuNP-LFIA	AuNPs	10^6^ (direct); 4 (after enrichment)	10 h enrichment+ <30 min	Bread, milk, jelly	[9]
Dual-mode LFIA (colorimetric/photothermal)	Ag–Au sea-urchin-like hollow nanospheres	2.4 × 10^3^ (colorimetric) 5.5 × 10^2^ (photothermal)	<30 min	Food samples	[10]
AIEgen-based LFIA	AIEFMs	3.06 × 10^2^	<30 min	Food samples	[11]
Dual-mode LFIA (colorimetric/fluorescent)	Dopamine-modified AuNPs	9.06 × 10^1^	<30 min	Food samples	[29]
Dual-signal enhanced LFIA	In situ growth of AuNPs + nanozyme catalytic deposition	1.25 × 10^1^	<30 min	Food samples	[30]
QD-based ICA	QDs + immunomagnetic separation	500	<1 h	Food	[39]

**Table 3:** Performance comparison of newly developed Immunological technologies for the detection of *E. coli* O157:H7.

Detection Technologies	Recognizing Elements	LOD (CFU/mL)	Linear Range (CFU/mL)	Actual Sample Recovery Rate	Ref.
Colorimetric	FeCoMOF/Co_3_O_4_@PDA	2	10^1^–10^8^	92.36–105.86%	[89]
	Fe_3_O_4_@TCPP@Pd	77	10^1^–10^6^	>95%	[90]
	Cu-AuNPs	Improve >40	10^3^–10^7^	>90%	[91]
	NPs/MOF-AgPt/PCN-223-Fe	276	10^3^–10^8^	91.56–118%	[92]
	HRP enzyme	50	10^2^–10^7^	92.5–107.3%	[26]
	BaTiO_3_/graphdiyne/Au	7	1–10^7^	89.0–112.6%	[93]
	CuSe nanoparticles	0.35 × 10^2^	10^2^–10^6^	93.97–103.47%	[94]
Fluoresence	mAb@R-CDs@BONs-NH_2_	25	10^1^–10^6^	92.3–106.8%	[95]
	CdSQDs@ZIF-8 MOFs	3	10^1^–10^8^	95.2–107.3%	[96]
	AIENPs	396	10^2^–10^7^	83.7–113.3%	[72]
	Time-resolved fluorescent microspheres	10^3^	10^3^–10^7^	98.5–102.3%	[97]
	FITC	10^4^	10^3^–10^7^	/	[98]
	AIE apta-sensor	2.8	6.5–6.5 × 10^7^	88.0–96.3%	[99]
PEC	UCNPs@SiO_2_@Ag/C-g-C_3_N_4_	2	5–5 × 10^6^	/	[64]
SERS-LFA	Fe_3_O_4_@Au-CP1 SERS	16	10^3^–10^6^	95.5–103.2%	[100]
ECL	Fe-Mn NCs	2.29	10^1^–10^8^	92.0–105.0%	[101]

## Data Availability

No new data were created or analyzed in this study.
